# IGFA Case Report of Pycnodysostosis Associated with Multiple Pituitary Hormone Deficiencies and Response to Treatment

**DOI:** 10.4274/jcrpe.galenos.2020.2019.0194

**Published:** 2020-11-25

**Authors:** Vishesh Verma, RK Singh

**Affiliations:** 1 Armed Forces Medical College, Department of Endocrinology, Pune, India; 2 Command Hospital, Clinic of Paediatrics, Lucknow, India

**Keywords:** Pycnodysostosis, short stature, multiple pituitary hormone deficiencies, Cathepsin-K gene mutation

## Abstract

Pycnodysostosis is a rare autosomal recessive osteosclerotic bone disorder associated with short stature and multiple bony abnormalities. Growth hormone (GH) deficiency may contribute to short stature in about 50% of patients. Available literature has rarely reported other pituitary hormone deficiencies in pyknodysostosis. Though the management remains conservative, recombinant human GH (rhGH) has been tried in selected patients. Here we present a case of pycnodysostosis which was evaluated for associated co-morbidities and found to have multiple pituitary hormone deficiencies. A 7-year-old girl was referred to our centre for evaluation of short stature. On examination, she had frontal and occipital bossing, limited mouth opening, hyperdontia with multiple carries, short and stubby digits and short stature. Investigation revealed dense sclerotic bones with frontal and occipital bossing, non-fusion of sutures with obtuse mandibular angle, non-pneumatised sinuses, small ‘J’ shaped sella turcica, acro-osteolysis of digits and absent medullary cavities. Cathepsin-K gene mutation analysis confirmed the diagnosis of pycnodysostosis. She was screened for associated co-morbidities and was found to have concomitant GH deficiency. Treatment with rhGH brought about an increase of 1 standard deviation score in height over 2 years and also unmasked central hypothyroidism at three months necessitating thyroxine replacement.

**What is already known on this topic?**

Pycnodysostosis is a rare autosomal recessive osteosclerotic bone disorder caused by a mutation in the Cathepsin-K gene. Growth hormone (GH) deficiency is associated with nearly half of the patients suffering from pycnodysostosis. The treatment is mainly conservative and supportive. Recombinant human GH (rhGH) has been used in a selected group of patients.

**What this study adds?**

We report a female with pycnodysostosis with associated with GH deficiency who was managed with rhGH. She had a favourable response with improvement in height standard deviation score of 1 over 18 months of treatment. rhGH unmasked central hypothyroidism requiring l-thyroxine replacement. A patient with pycnodysostosis requires monitoring for central hypothyroidism and other pituitary hormone deficiencies, especially if rhGH treatment is being offered.

## Introduction

Pycnodysostosis is a rare autosomal recessive osteosclerotic bone disorder (1). It is caused by homozygous or compound heterozygous mutation in the *Cathepsin-K (CTSK)* gene, which maps to chromosome 1q21 (2,3,4). The disease is characterised by specific bony abnormalities, facial features and short stature (5,6). Growth hormone (GH) deficiency is present in about half the patients with pycnodysostosis (5). However, other pituitary hormone deficiencies have not been reported to date. 

Presently, there is no established therapy for pycnodysostosis (7,8). The management is primarily symptomatic and preventive (9). In a case series of eight patients with pycnodysostosis, four patients had associated GH deficiency. These four responded well to GH treatment with normalisation of insulin-like growth factor-1 (IGF-1) and acceleration in growth velocity (5). Karamizadeh et al (10) in a cohort of a further eight patients with pycnodysostosis reported a positive impact on linear growth with GH treatment. GH treatment, based on an IGF-1-based dosing regimen, when offered to three children with pycnodysostosis and 16 children of idiopathic short stature, resulted in near-normal stature and body proportion (11). Genetic testing and counselling is often a neglected aspect in pycnodysostosis and should be offered to all patients and relatives.

Here we present a case of pycnodysostosis associated with GH deficiency. The patient responded favourably to recombinant human GH (rhGH) treatment. However, rhGH treatment unmasked central hypothyroidism, necessitating l-thyroxine replacement. 

## Case Report

**Figure 1:**
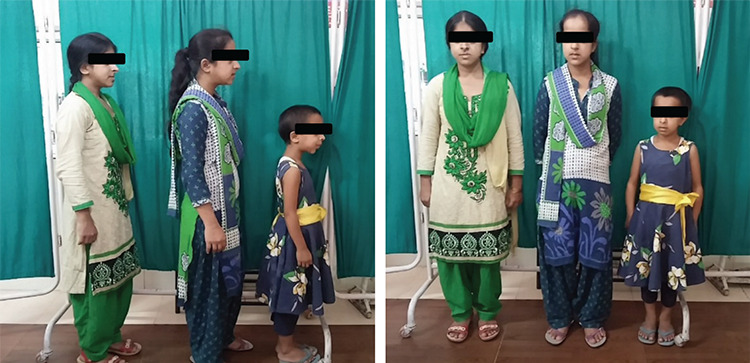
The index case (extreme right) and the sibling sister aged 16 years (centre) and 18 years (extreme left) share the same phenotypic features

**Figure 2:**
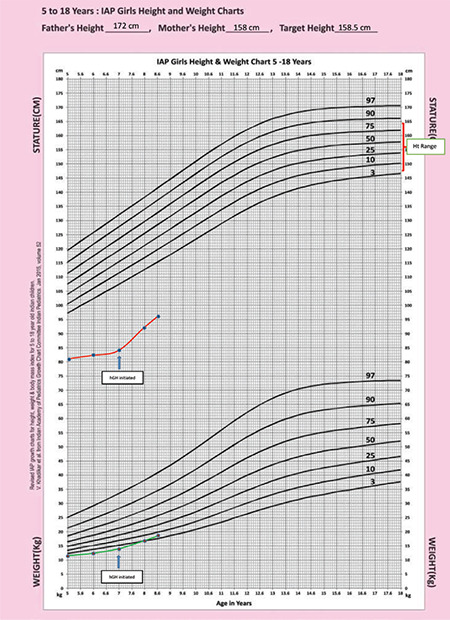
Growth chart of the index patient showing increase in height velocity on starting treatment

**Figure 3:**
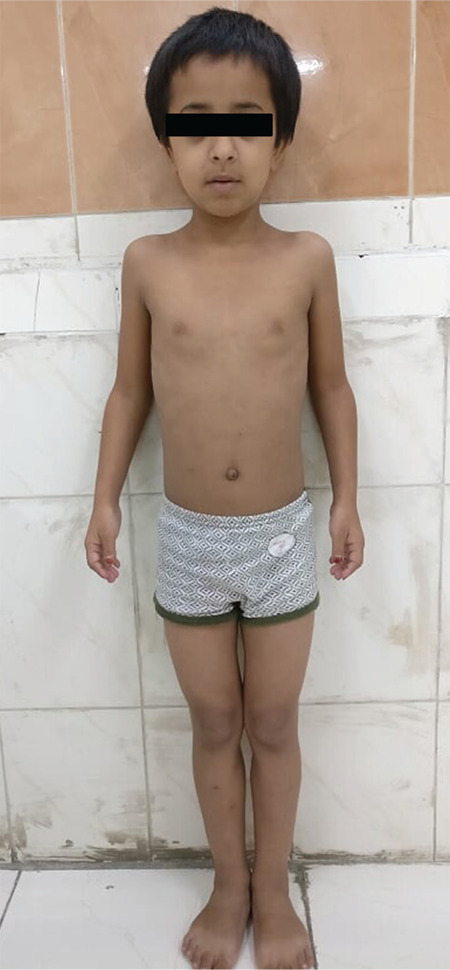
Clinical image of the index patient. Frontal bossing and typical facies are present. The carrying angle is wide

**Figure 4:**
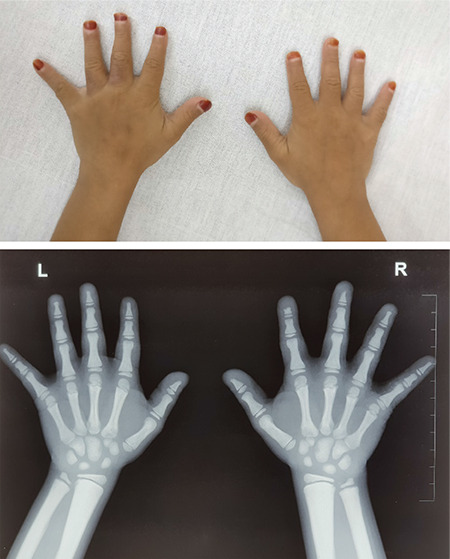
The image shows short and stubby digits. X-ray reveals acro-osteolysis of the terminal phalanges. The medullary cavity is absent in the long bones of the hand

A 7-year-old girl was referred to our centre for evaluation of short stature. She was the product of a second-degree consanguineous marriage. She had two elder sisters, aged 16 and 18 years, who had similar morphological features and a brother aged 14 who was healthy (Figure 1). The sisters had attained menarche at 14 years of age. Anti-natal and peri-natal periods were uneventful. Her birth weight was 2.1 Kg. The parents reported a poor gain in height since birth. She had a history of two fractures on trivial trauma; one involving the right tibia and one involving the left radius at 4 and 5 ½ years of age respectively. The fractures took a long time to heal. Height and weight were plotted on the Indian Association of Paediatrics growth chart for Indian girls aged 5-18 years (12). On physical examination, her height was 84 cm [-5.6 standard deviation score (SDS)], and the head circumference was 68.5 cm (Figure 2). The mother’s height was 158 cm, and the father’s height was 172 cm, thus her target height was 158.5 cm. The affected sisters at 16 and 18 years of age were 110.5 and 114 cm, respectively and both were below the 5th centile. The height of the unaffected elder brother was 158 cm, approximating to the 50th centile. The brother had not reached a final height. The sibling sisters have attained final height, as the epiphyses were fused. Notably, the final heights of the sibling sisters was less than expected, even for patients with Pycnodystosis. The medical records of the index case showed that her height velocity had been 1.2 cm/year for the last two years. She was pre-pubertal. She had facial dysmorphism with frontal, occipital bossing and limited mouth opening (Figure 3). She had short hands with short and stubby fingers with dystrophic nails (Figure 4). Her anterior fontanelle had not closed and measured 1.8 cm. The fontanelles of the affected sisters had closed. Examination of the oral cavity revealed hyperdontia, multiple caries and a grooved palate. Hepatosplenomegaly was present.

**Figure 5:**
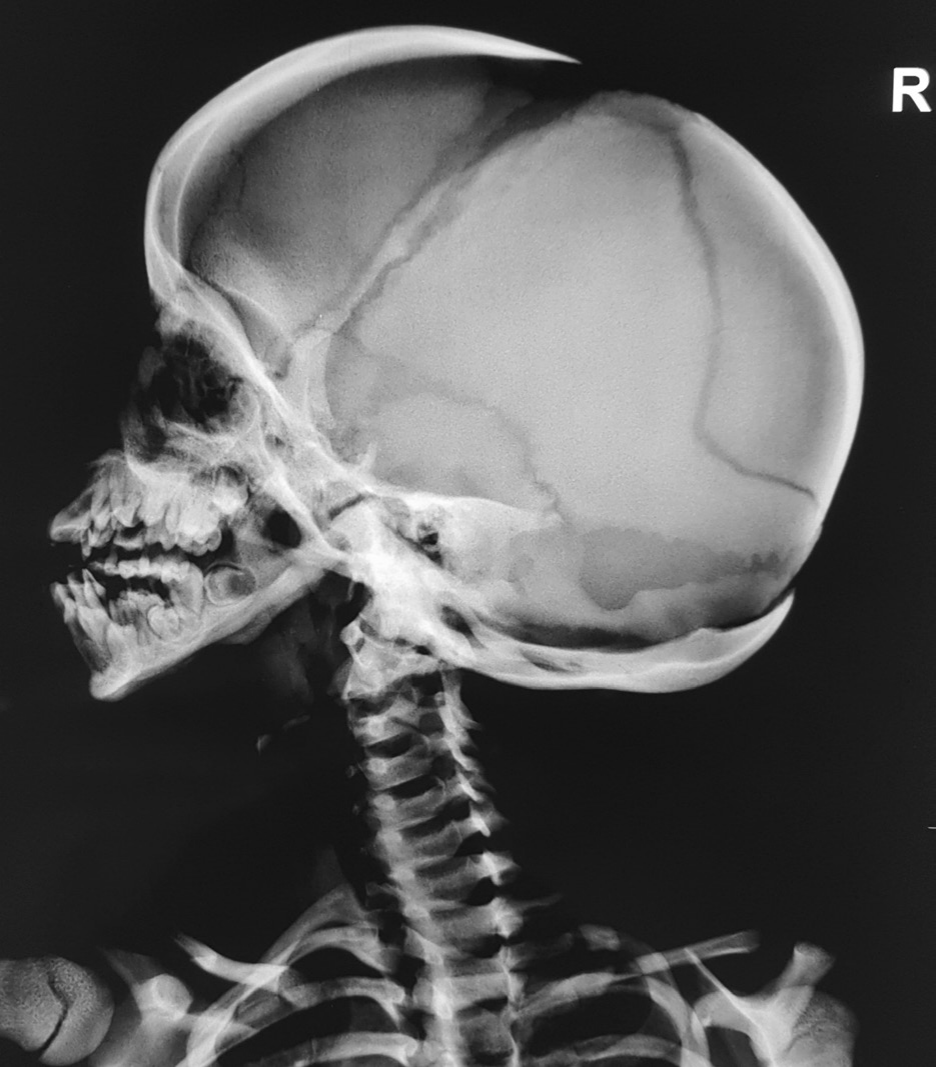
X-ray skull lateral view showing open fontanelles, obtuse mandibular angle and non-pneumatised sinuses. Also noted is acro-osteolysis of the clavicle

Laboratory examination revealed normal haematological parameters. The serum calcium, urea, creatinine, phosphorus, alkaline phosphatase, intact parathyroid hormone and 25 (OH) vitamin D levels were normal. Her IGF-1 level was 56 µg/L (normal range: 58-367 µg/L for a seven-year-old girl). A GH stimulation test was carried out with clonidine and revealed a peak value of 1.1 ng/mL, indicating GH deficiency. Her basal and adrenocorticotropin hormone stimulated cortisol levels were within normal limits. Serum thyroxine (T4) was 6.5 µg/dL (normal value: 4.6-12 µg/dL), and TSH was 3.2 mIU/mL. Serum IGF-1 for the affected sister aged 16 years was 182.6 µg/L (normal range: 127-541 µg/L) and of the sister aged 18 years was 201.2 µg/L (normal range: 121-486 µg/L); both had normal thyroid function profiles. Radiographic examination revealed generalised osteosclerosis. The medullary cavities were absent in the long bones. Skull radiography revealed frontal and occipital bossing, non-fusion of sutures with obtuse mandibular angle, absence of mastoid air cells and small ‘J’ shaped sella turcica (Figure 5). Terminal phalanges of hands showed acro-osteolysis (Figure 4). Magnetic resonance imaging of the sella revealed a hypoplastic anterior pituitary with a volume of 121 mm^3^ (<5^th^ centile for age) (13) and a typical posterior pituitary bright spot. Arterial blood gas analysis did not reveal any hypoxia. Polysomnography did not show any evidence of obstructive sleep apnoea. Audiometry and ophthalmic examination were normal.

Molecular testing of the *CTSK* gene showed a homozygous missense variant CTSK:C.890G>C. The parents were found to be carriers of the same variant. Consent could not be obtained from the affected sisters and the unaffected brother for genetic testing due to the financial constraints of the family. The parents were counselled regarding their carrier status, and the siblings were advised regarding the benefits of genetic testing.

The index case was offered conservative and symptomatic management. Orthodontic and endodontic treatment was provided for caries and malposition of teeth. Counselling regarding oral hygiene, fracture prevention and other psychiatric aspects of the disease were undertaken.

In light of the GH deficiency, rhGH was administered at 0.16 mg/kg/week. An IGF-1 level was repeated after four weeks, which was still low. Based on the IGF-1 response, the dose was gradually increased to 0.48 mg/kg/week. An incremental IGF-1 response and increase in height velocity ruled out GH resistance. Compliance with treatment was good on regular monitoring. On follow-up, a repeat evaluation of her thyroid axis after three months of treatment revealed a T4 of 3.1 µg/dL and TSH of 9.2 mIU/mL. She was started on levothyroxine replacement of 50 µg/day and after two months of replacement therapy the T4 had normalised to 10.4 µg /dL and the TSH was 4.9 mIU/mL. At the end of 18 months of treatment, her height was 97 cms with a 9 cm increase in stature over the first year of treatment and four cms over the next six months. Her height velocity was 8.7 cm/year over 18 months, and there was a gain of 1 SDS in height over this treatment period. There was no adverse effect on GH treatment.

Informed consent was obtained from the guardian.

## Discussion

Pycnodysostosis remains a rare cause of short stature. However, in patients suffering from the disease, short stature is a constant feature, afflicting 90.32% of cases ranging from -1.5 to -6 SDS, with our case having a height SDS of -5.6 at presentation (13). The short height is primarily due to impaired bone remodelling and subsequent sclerosis of the bones. Other contributors to short stature in pycnodysostosis are malnutrition, chronic airway obstruction and hypoxemia (5). Our patient had a healthy body mass index and arterial blood gas analysis, and so these abnormalities were ruled out in our patient. Pycnodysostosis may be associated with hypopituitarism (4). In our patient, the pituitary-adrenal axis was intact. However, she had a low IGF-1. As alteration in the GH-IGF-1 axis has not been studied in pycnodysostosis, we decided to confirm GH secretory defect with a GH stimulation test, with an inadequate secretory response indicating GH deficiency. GH deficiency has been demonstrated in 50% of the patients of pycnodysostosis. The deficiency may be due to pituitary hypoplasia caused by an increased bone volume of the sella and increased intrasellar pressure (5). Bone age in pycnodysostosis cannot be accurately assessed as disease-specific nomograms are not available making bone age determination difficult to assess in these patients.

The diagnosis is clinical and is based on relevant history, clinical features and radiological examination, as was in this case. If available, a genetic analysis should be carried out. We could demonstrate a *CTSK* gene homozygous missense variant CTSK:C.890G>C on molecular genetic testing. The mutation has been previously reported in humans (HGMD CS072172). The mutation results in a change in protein structure with threonine replacing serine at position 297 of the Cathepsin-K protein (S297T) (14). Polyphen-2 predicts the mutation to be “Possibly damaging” and Mutationtaster predicts the mutation to be “Damaging” (15,16). Genetic analysis may reveal novel *CTSK* gene mutation in a homologous or a compound heterozygous pattern (2). Genetic testing, and counselling is often a neglected aspect of the disease. Genetic testing was offered to the index patient and the parents. However, due to financial constraints, this offer could not be extended to the siblings.

The treatment remains conservative and primarily targets preventive counselling. The patients can live a near-normal life. GH and IGF-1 have an anabolic role in bone metabolism (17). Only a few studies have demonstrated the efficacy of rhGH treatment for pycnodysostosis. Rothenbühler et al (11) initiated GH treatment in three children with pycnodysostosis at a dose of 29 µg/kg/day, 67 µg/kg/day and 120 µg/kg/day and observed near-normal adult stature and normalisation of skeletal proportions. GH treatment, at a dose of 18 U/m2/week, significantly improved growth velocity from 3.3±0.8 cm/year to 9.4±2.1 cm/year during the first year and 7.5±1 cm/year in the second year (5). Height SDS and growth velocity increased on rhGH treatment at a dose of 50 µg/kg/day, when compared to pre-treatment levels and after stopping GH therapy in patients manifesting with GH deficiency in pycnodysostosis (10). The dose used by our case was initially 23 µg/kg/week, which was gradually increased to 68 µg/kg/week based on IGF-1 levels. The doses of rhGH required for pycnodysostosis are reported to be higher than those used in idiopathic short stature and GH deficiency (18), and this was true in our patient (5,10,11). Our patient had an increase in growth velocity to 8.7 cm/year. The SDS also improved from -5.6 to -4.59, a gain of 1 SDS. rhGH treatment may unmask central hypothyroidism in between 36% and 47% of patients who appear euthyroid before initiation of rhGH treatment (19). Central hypothyroidism, though rarely reported in pycnodysostosis, was exposed by rhGH therapy in our patient, requiring levothyroxine replacement.

## Conclusion

Pycnodysostosis remains a rare cause of short stature and is associated with various skeletal and dental abnormalities. GH deficiency may be present in around half of these patients. Other contributors to short height including malnutrition, chronic hypoxemia, hypopituitarism and intrinsic short stature should be evaluated in all patients. Though the primary management remains conservative, GH treatment is effective in some patients. Other pituitary hormone deficiencies may be associated with pycnodysostosis, and rhGH treatment may unmask underlying central hypothyroidism in this group of patients. Genetic counselling should be offered to all patients and their family.

## References

[1] Gelb BD, Shi GP, Chapman HA, Desnick RJ (1996). Pycnodysostosis, a lysosomal disease caused by cathepsin K deficiency. Science.

[2] Turan S (2014). Current research on pycnodysostosis. Intractable Rare Dis Res.

[3] De Ridder R, Boudin E, Mortier G, Van Hul W (2018). Human genetics of sclerosing bone disorders. Curr Osteoporos Rep.

[4] Motyckova G, Fisher DE (2002). Pycnodysostosis: role and regulation of cathepsin K in osteoclast function and human disease. Curr Mol Med.

[5] Soliman AT, Ramadan MA, Sherif A, Aziz Bedair ES, Rizk MM (2001). Pycnodysostosis: clinical, radiologic, and endocrine evaluation and linear growth after growth hormone therapy. Metab Clin Exp.

[6] Aynaou H, Skiker I, Latrech H (2016). Short stature revealing a pycnodysostosis: a case report. J Orthop Case Rep.

[7] Mujawar Q, Naganoor R, Patil H, Thobbi AN, Ukkali S, Malagi N (2009). Pycnodysostosis with unusual findings: a case report. Cases J.

[8] Chavassieux P, Asser Karsdal M, Segovia-Silvestre T, Neutzsky-Wulff AV, Chapurlat R, Boivin G, Elmas PD (2008). Mechanisms of the anabolic effects of teriparatide on bone: insight from the treatment of a patient with pycnodysostosis. J Bone Miner Res.

[9] Bizaoui V, Michot C, Baujat G, Amouroux C, Baron S, Capri Y, Cohen- Solal M, Collet C, Dieux A, Geneviève D, Isidor B, Monnot S, Rossi M, Rothenbuhler A, Schaefer E, Cormier-Daire V (2019). Pycnodysostosis: Natural history and management guidelines from 27 French cases and a literature review. Clin Genet.

[10] Karamizadeh Z, Ilkhanipoor H, Bagheri F (2014). Effect of growth hormone treatment on height velocity of children with pycnodysotosis. Iran J Pediatr.

[11] Rothenbühler A, Piquard C, Gueorguieva I, Lahlou N, Linglart A, Bougnères P (2010). Near normalization of adult height and body proportions by growth hormone in pycnodysostosis. J Clin Endocrinol Metab.

[12] Khadilkar VV, Khadilkar AV (2015). Revised Indian Academy of Pediatrics 2015 growth charts for height, weight and body mass index for 5-18-year-old Indian children. Indian J Endocrinol Metab.

[13] Fink AM, Vidmar S, Kumbla S, Pedreira CC, Kanumakala S, Williams C, Carlin JB, Cameron F (2005). Age-related pituitary volumes in prepubertal children with normal endocrine function: volumetric magnetic resonance data. J Clin Endocrinol Metab.

[14] Homo sapiens cathepsin K (CTSK), mRNA. Available at: (cited 2020 Jan 18).

[15] PolyPhen-2: prediction of functional effects of human nsSNPs. Last accessed date: 2020 Jan 19. Available at:.

[16] MutationTaster. Last accessed date: 2020 Jan 19. Available at:.

[17] Locatelli V, Bianchi VE (2014). Effect of GH/IGF-1 on Bone Metabolism and Osteoporsosis. Int J Endocrinol.

[18] Grimberg A, DiVall SA, Polychronakos C, Allen DB, Cohen LE, Quintos JB, Rossi WC, Feudtner C, Murad MH;, Drug and Therapeutics Committee and Ethics Committee of the Pediatric Endocrine Society (2016). Guidelines for Growth hormone and insulin-like growth factor-I treatment in children and adolescents: growth hormone deficiency, idiopathic short stature, and primary insulin-like growth factor-I deficiency. Horm Res Pediatr.

[19] Behan LA, Monson JP, Agha A (2011). The interaction between growth hormone and the thyroid axis in hypopituitary patients. Clin Endocrinol (Oxf).

